# Surgical Start Time Impact on Hospital Length of Stay for Elective Inpatient Procedures

**DOI:** 10.7759/cureus.16259

**Published:** 2021-07-08

**Authors:** Christopher P Cifarelli, John P McMichael, Alex G Forman, Paul A Mihm, Daniel T Cifarelli, Mark R Lee, Wallis Marsh

**Affiliations:** 1 Neurological Surgery, West Virginia University School of Medicine, Morgantown, USA; 2 Radiation Oncology, West Virginia University School of Medicine, Morgantown, USA; 3 Information Technology, Cleveland Clinic, Cleveland, USA; 4 Strategic Analytics, West Virginia University School of Medicine, Morgantown, USA; 5 Surgical Services, West Virginia University School of Medicine, Morgantown, USA; 6 Neurosurgery, West Virginia University School of Medicine, Morgantown, USA; 7 Surgery, West Virginia University School of Medicine, Morgantown, USA

**Keywords:** length of stay, elective surgery, surgery start time, after-hours care, surgical scheduling

## Abstract

Background

Hospital length of stay (LOS) remains an important, albeit nonspecific, metric in the analysis of surgical services. Modifiable factors to reduce LOS are few in number and the ability to practically take action is limited. Surgical scheduling of elective cases remains an important task in optimizing workflow and may impact the post-surgical LOS.

Methods

Retrospective data from a single tertiary care academic institution were analyzed from elective adult surgical cases performed from 2017 through 2019. Emergent or urgent add-on cases were excluded. Variables included primary procedure, age, diabetes status, American Society of Anesthesiologists (ASA) class, and surgical start time. Analysis of the median LOS following surgery was performed using Mann-Whitney tests and Cox hazards model. Matched-cohort analysis of mean total hospitalization costs was performed using the Student’s t-test.

Results

9,258 patients were analyzed across five surgical service lines, of which 777 patients had surgical start time after 3 PM. The median LOS for the after 3 PM group was 1 day longer than the before 3 PM start time cohort (3.0 vs 2.1, p < 0.001). Service line analysis revealed increased LOS for Orthopedics and Neurosurgery (3.0 vs 1.9, p < 0.001; 3.0 vs 2.0, p < 0.05). Multivariate analysis confirmed that start time before 3 PM predicted shorter LOS (HR = 1.214, 1.126-1.309; p < 0.001). Case-matched cost analysis for frequently performed orthopedic and neurosurgical cases with an after 3 PM start time failed to demonstrate a significant difference in total hospital charges.

Conclusion

Optimization of surgical services scheduling to increase the proportion of elective surgical cases started before 3 PM has the potential to decrease post-surgical LOS for adult patients undergoing Orthopedic or Neurosurgical procedures.

## Introduction

The operational efficiency of elective surgical procedures with planned hospital admissions is dependent on multitude of factors, only a small portion of which are modifiable. Patient age, comorbid states, surgical experience of the operative staff, and the underlying diagnosis are all areas in which the patients and providers have limited control. Conversely, parameters such as the choice of peri-operative medications, post-operative mobilization plans and surgical start time are slightly more adaptable and offer the potential opportunity for optimization. 

Hospital length of stay (LOS) remains one of the most studied endpoints in outcomes research as it relates to efficiency in care delivery. Many studies have focused on the use of opioids, glycemic levels, and cardiac function in the development of predictive models, usually as they relate to specific surgical procedures [1]. From a resource utilization standpoint, LOS variation impacts the ability to offer elective and non-elective services to additional patients, while also fiscally creating burdens for services with fixed reimbursement schedules, regardless of the numbers of hospitalization days [2]. 

Attempts to transition appropriate elective surgical procedures from hospital admission status to same day surgery have been moderately successful from the perspectives of both patients and healthcare delivery systems [3-5]. Unfortunately, a significant proportion of elective surgeries, especially those involving orthopedic or cancer care, still require some form of inpatient hospitalization. Recent stresses in the U.S. healthcare system as related to the COVID-19 pandemic have exacerbated the need for timely hospital discharge for elective surgical patients based on system throughput and exposure risk. As many healthcare systems look to expand the use of non-traditional operative days (weekends) and times of day (after 3 PM start times) to compensate for access limitations early in the pandemic, there is an unknown in the impact that such modifications will have to surgical outcomes, specifically hospital LOS. 

The current study reviews the single-institutional elective surgical experience of a large academic medical center that serves a rural patient population spread over approximately 25,000 square miles for a three-year period between 2017 and 2019 with analysis of hospital LOS as a function surgical start time. These data have been previously discussed in preprint format (DOI: 10.21203/rs.3.rs-94936/v1) prior to peer-review publication. 

## Materials and methods

Data inquiries were made of the central surgical scheduling database and hospital charge master dataset for elective adult surgical cases performed at West Virginia University Ruby Memorial Hospital from 2017 through 2019. WVU IRB exemption was granted non-human-subject research. De-identified data were coded based on age at the time of surgery, ASA class, diabetes status, surgical start time based on anesthesia release, length of post-surgical hospital stay and type of surgical service performing the procedure. Cases involving patients under 18 years of age, emergency/urgent non-elective status, post-surgical hospitalizations greater than 90 days, planned same-day surgeries, or weekend elective cases were excluded from the current analysis. In addition, patients who expired during hospitalization for an elective procedure were not included in the data set. Surgical start times were dichotomized as pre- and post-3 PM based on the institutional pattern of standard daytime change of shift that affects more than 75% of operating room personnel.

Univariate analysis of the effect of surgical start time was performed using the non-parametric Mann-Whitney test based on the non-normal distribution of post-surgical hospital length of stay data. Multi-variate analyses were performed according to the Cox proportional hazard model using variables of age (<65 years), diabetic status, American Society of Anesthesiologists (ASA) classification, and surgical start time. Proportional hazard modeling was utilized based on the potential of any of the selected parameters to impact the length of hospitalization and time to event was defined as admission length of stay from operating room discharge to facility discharge. Case matching for total hospitalization charge analysis was performed based on primary procedural CPT code, patient age at the time of surgery, and gender. Cost analysis was based on charges only, not absolute costs or reimbursement, and performed on cases where the patient was admitted for hospitalization on the day of the procedure. In order to eliminate the existence of pre-surgical hospital charges during the admission, only cases admitted on the day of surgery were included. All statistical analyses were performed using Statistical Package for the Social Sciences (SPSS) v.26 (IBM Corp, Armonk, NY) with significance at p < 0.05. 

## Results

Patient demographics

Between 2017 and 2019, a total of 9,258 adult elective surgical cases were performed amongst five primary surgical service lines, including General Surgery, Surgical Oncology, Orthopedic Surgery, Cardiac Surgery, and Neurological Surgery (summarized in Table 1). 

**Table 1:** Case demographics by surgical service line. The total number of cases performed between 2017 through 2019 on adult patients (18 years and older) with a planned elective post-operative admission were stratified according to surgical start time, age, diabetes status, and American Society Anesthesiologist (ASA) classification.

	Total cases	Start time	Start time	Mean age (±SEM)	Median age	Diabetic patients	ASA classes
		Before 3 PM	After 3 PM			(% of total)	(% of total)
General Surgery	1723	1546	177	49.8 ± 0.373	50	411 (23)	I: 7 (0.4)
							II: 178 (10)
							III: 1271 (75)
							IV: 224 (13)
Orthopedic Surgery	4335	4061	274	59.9 ± 0.220	62	726 (17)	I: 63 (1.5)
							II: 1010 (24)
							III: 2952 (70)
							IV: 206 (5)
Surgical Oncology	1261	1163	98	58.8 ± 0.418	60	204 (16)	I: 5 (0.4)
							II: 206 (17)
							III: 937 (76)
							IV: 92 (8)
Neurological Surgery	1640	1427	213	55.5 ± 0.375	57	268 (16)	I: 10 (0.6)
							II: 292 (18)
							III: 1182 (73)
							IV: 137 (9)
Cardiac Surgery	299	284	15	58.8 ± 0.862	61	72 (24)	I: 0 (0)
							II: 5 (2)
							III: 67 (27)
							IV: 178 (71)
Total	9258	8481	777	57.1 ± 0.159	59	1681 (18)	I: 85 (1)
							II: 1691 (19)
							III: 6409 (71)
							IV: 837 (9)

The greatest proportion of cases were attributed to the Orthopedic Surgery service, with 4335 cases comprising 46% of the total cases analyzed. The median age was 59 years at the time of surgery, with 1681 patients (18%) having an admission diagnosis of diabetes mellitus. The American Society of Anesthesiologists (ASA) Physical Status Classification System rating were applied to each patient at the time of admission, with over 70% of total patients receiving an ASA III class. This trend was preserved across all service lines, except Cardiac Surgery, where 71% of patients were designated ASA IV. The surgical start time was coded according to cases starting before or after 3 PM, with 8,481 (92%) of cases starting before 3 PM. 

Hospital length of stay based on surgical start time

The percentage of cases with start time before 3 PM ranged from 87% to 95% according to service line, with Orthopedic and Cardiac Surgery preserving the highest percent of total cases with before 3 PM commencement (94% and 95%, respectively) (Table 2). 

**Table 2:** Hospital length of stay (LOS) based on surgical start time. The mean and median LOS were compared for all cases and within each service line.  Statistical differences in median LOS were determined by the Mann-Whitney U test based on the non-normal distribution of LOS data with significance noted at p < 0.05.  Both Orthopedic and Neurological services demonstrated and an increase in median LOS of 1 day for cases started after 3 PM.

	Before 3 PM start time (%)	After 3 PM start time (%)	Median LOS	Mean LOS	p
			Before 3 PM	Before 3 PM	
			After 3 PM	After 3 PM	
General Surgery	1546 (88)	177 (10)	2.0	5.1	0.768
			1.9	7.6	
Orthopedic Surgery	4061 (94)	274 (6)	1.9	3.5	<0.001
			3.0	6.3	
Surgical Oncology	1163 (92)	98 (8)	3.9	5.7	0.606
			4.5	5.5	
Neurological Surgery	1427 (87)	213 (13)	2.0	4.6	<0.05
			3.0	5.4	
Cardiac Surgery	284 (95)	15 (5)	6.1	9.9	0.324
			5.7	10.3	
Total	8481 (92)	777 (8)	2.1	4.5	<0.001
			3.0	6.3	

The median post-surgical length of stay for all cases was 2.1 days for the before 3 PM group and three days for the after 3 PM group (p < 0.001). Mean values for these same groups remained shorter for the before 3 PM start time group compared to the after 3 PM group, although the non-normality of the LOS data precluded the ability to accurately perform a statistical test based on the means, with a skewness value of 4.78 ± 0.025. Of the service lines examined, Orthopedic Surgery and Neurological Surgery were both found to have a significantly shorter median LOS in the before 3 PM start time group based on the Mann-Whitney Test (U = 413072, p < 0.001; U = 139251, p < 0.05, respectively). 

Predictors of post-surgical length of stay

Semi-parametric multivariate analysis revealed predictors of post-operative length of stay according to the Cox proportional hazards model, including age (<65 years), ASA class (I-IV), diabetes mellitus status, and surgical start time (before 3 PM) were used in the modeling. Hazard ratios (HR) demonstrated significance for shorter post-surgical length of stay based on age <65 (HR = 1.046, 95%CI 1.001-1.093), absence of a diabetes mellitus diagnosis (HR = 1.137, 95% CI 1.077-1.20), and surgical start time before 3 PM (HR = 1.214, 95%CI 1.126-1.309) (Table 3).

**Table 3:** Cox proportion hazard model of predictors of LOS. Age, ASA classification, diabetes status, and surgical start times were dichotomized and analyzed using Cox regression methodology.  Hazard ratios (HR, Exp(B)) and the associated 95% confidence intervals (CI) were expressed with p < 0.05 in all categories. Positive predictors of a shorter LOS included age <65 years, non-diabetic status and surgical start time before 3 PM.

			95% CI for Exp (B)		
		HR Exp (B)	Lower	Upper	p
Age (<65 years)		1.046	1.001	1.093	<0.05
ASA	1	0.134	0.033	0.544	0.005
	2	0.129	0.032	0.517	<0.005
	3	0.096	0.024	0.386	0.001
	4	0.048	0.012	0.194	<0.001
Non-diabetic		1.137	1.077	1.20	<0.001
Start time before 3 PM		1.214	1.126	1.309	<0.001

Surgical case categories for orthopedic surgery & neurological surgery

The identification of increased LOS in patients with surgical start time after 3 PM in the Orthopedic and Neurological service lines prompted an evaluation of the case mixtures as defined by body location in Orthopedics and sub-specialty in Neurological surgery. Based on primary CPT codes, the majority of adult elective orthopedic surgery cases performed after 3 PM were leg/ankle cases (45%) and evenly distributed among the other anatomical sites including pelvis/hip, femur/knee, arm/elbow, and spine, ranging from 8.2% to 13% (Table 4). 

**Table 4:** Orthopedic and neurosurgical case categories. Orthopedic cases were organized based on anatomic region and stratified according to surgical start time. Neurosurgical cases were divided into cranial, spinal, and peripheral/functional cases and stratified by start time.

Orthopedic cases by start time			
Surgical site	Before 3 PM N=	After 3 PM N= (% of total after 3 PM)	Total N=
Pelvis/hip	1406	22 (8.2%)	1428
Femur/knee	1046	30 (11.2%)	1076
Leg/ankle	387	122 (45.4%)	509
Foot	93	7 (2.6%)	100
Shoulder/humerus	54	8 (3.0%)	62
Arm/elbow	97	35 (13.0%)	132
Hand	14	1 (0.4%)	15
Spine	770	32 (11.9%)	802
Neurosurgical cases by start time			
Cranial	651	98 (48.3%)	749
Spine	673	99 (48.8%)	772
Peripheral/functional	45	6 (3.0%)	51

Within Neurological Surgery, the mixture of cranial versus spine cases performed after 3PM was even at 48% each, with peripheral nerve/functional cases only representing 3% of late start cases.

Total hospital charge analysis for orthopedic surgery & neurological surgery

The three highest frequency cases in Orthopedics were total hip arthroplasty (CPT 27130, n = 12), arthroplasty knee revision (CPT 27486; n = 17) and arthroplasty knee total (CPT 27447; n = 14). Within Neurological Surgery, the highest frequency cases were cranioplasty (CPT 62148; n = 7), craniotomy for tumor (CPT 61546; n = 9), and anterior cervical discectomy and fusion (CPT 22551; n = 15) (Table 5). 

**Table 5:** Mean total hospital charge difference based on surgical start time. Cases without precedent hospitalization relative to the primary surgical procedure were matched between the before 3 PM and after 3 PM start time cohorts.  The three most frequent procedures based on CPT code were compared for differences in mean total hospital charges. No statistically significant differences were noted between the two groups (p > 0.05).

CPT primary procedure	Before 3 PM start mean total charges	After 3 PM start mean total charges	p
Orthopedic Surgery			
27130 Arthroplasty Total Hip	48,306	53,880	0.49
27486 Arthroplasty Knee Revision	85,176	86,591	0.87
27447 Arthroplasty Knee Total	40,540	50,644	0.09
Neurological Surgery			
62148 Cranioplasty	40,782	50,412	0.14
61546 Craniotomy for Tumor	109,212	85,729	0.14
22551 Discectomy Spine Anterior Cervical with Fusion	53,541	51,889	0.82

No statistically significant differences were found (p > 0.05) among cases matched based on CPT code, age, or gender, nor were any consistent trends noted, with mean total charges being slightly higher in the after 3 PM group within Orthopedic Surgery and for the cranioplasty procedure within the Neurological Surgery case matched cohorts. 

## Discussion

Value propositions in healthcare remain at the forefront of both patient and provider interests. To that end, government agencies, private insurers, and patient advocacy groups have all spent considerable time and effort in attempting to define reliable metrics that are reflective of optimal value in care. Perhaps the most simplified measure of value for all involved in delivering and receiving healthcare service is time. Although lacking in specific information regarding outcome and/or satisfaction, hospital length of stay has been the predominant measure in outcomes research, largely based on the universal understanding that the reduction in hospitalization time is a positive attribute.

The impact of elective surgical procedures on overall operating costs for US hospitals was highlighted by the Healthcare Cost and Utilization Project published through the Agency for Healthcare Research and Quality (AHRQ) where only 29% of hospitalizations involved a surgical procedure, but these hospitalizations accounted for 48% of the $387 billion in hospital costs in 2011 [6]. Of interest, this study also concluded that hospital admissions involving OR procedures were associated with a longer mean length of stay (5.0 vs 4.4 days) compared to admissions without surgery and the percent of elective surgical admissions was more than 3-fold higher than non-surgical admissions (48% vs 13% of total N). Overall, this work demonstrated the significant proportion of expenditure and resource utilization accompanying surgical admissions in the US, while alluding to the need to identify areas for cost reduction. 

Elective surgical procedures present a number of opportunities for augmentation in the need for inpatient hospitalizations. Compared to urgent or emergent procedures, the elective surgeries allow for a controlled approach to post-surgical disposition planning and a reduction in patient/family anxiety. From a system occupancy perspective, the transition away from universal overnight hospitalization for short procedures to same-day surgery has been seen in a multitude of areas [7-9]. Yet, the ability to transition an overnight observation case to a same-day surgery case is not universal, regardless of the case complexity. In the setting of anterior cervical discectomy and fusion, a common spine procedure that has seen a trend toward same-day discharge, Mayo et al found that the time of day of the procedure was predictive of the ability to discharge same day. In that study, a “late” mean surgical start time of 12:19 (range: 9:10-17:51) resulted in a higher likelihood of requiring overnight hospitalization (HR 1.6±0.30; p = 0.010) [10]. Of course, the limitations of same-day discharge in this situation is affected by mandatory minimum post-anesthetic observation time, unlike the cases in which overnight admission is guaranteed post-operatively. 

In the current study, we identify a significant LOS effect among elective cases with planned hospital admissions in which surgical start time is documented after 3 PM, which accounts for 8.4% of all 9258 cases analyzed. Based on service line demarcation, only Orthopedic and Neurological surgery appeared to be effected, with these cases representing 65% of the total cases analyzed. Unfortunately, a more detailed analysis of these cases based on surgical site, failed to demonstrate a significant predominance of a particular procedure that could account for the time of day effect. In fact, the distribution in Neurosurgical cases between cranial and spine was precisely even at 48% of all after 3 PM cases within that service line, providing an argument against the impact of bias in case scheduling. While the multivariate analysis of age, ASA classification, and diabetes status all found significant predictors of shorter LOS in the non-diabetic, ASA Class I patient under the age of 65, the start time before 3 PM was the most powerful indicator (HR 1.214; p < 0.001).

As with most modern value assessments in healthcare, total hospital charges accrued during an episode of care remain an important metric. Continued evolution of the reimbursement process will likely increase the importance of total charges for all hospital systems, with potential likelihood that fixed payment schedules may eventually become universal based on diagnostic code [11]. Experiences with bundled payments in orthopedic and spinal procedures has resulted in mixed response with respect to total episode costs, without differences in length of stay [12]. Our analysis failed to demonstrate a significant difference in case-matched charges for procedures performed in the before or after 3 PM cohorts, although the trend toward higher charge accumulation followed with the increased LOS in the after 3 PM for four out of the six procedures analyzed. 

The demarcation of the 3 PM start time was not arbitrary, but based on the inherent shift changes that impact operating room staff, including nursing, surgical technologists, and anesthetists. Given the mixture of room personnel, there was the highest likelihood that at least one team member transitions off shift and is replaced by another that was not present for the start of the case. In addition, previous literature has supported the concept that adverse anesthetic effects are more likely to occur in patients with surgical start time after 3 PM [13]. Similarly, anesthetic handoffs are more likely during evening or weekend cases in a large series analysis of the ACS NSQIP, where documented post-operative complications are more likely, although no causal relationship has been established, nor is the presence of a handoff predictive of a post-surgical morbidity after correcting for the co-variate of time of day [14].

Our data is contrasted to that of many other studies that examine the time of day effect on procedural safety, namely in the overall survival or development of co-morbidities, such as infection, cardiac event, or stroke [15-18]. With respect to morbidities, the literature is dichotomous with some studies dismissing the impact of surgical start time, while others underscoring the importance of such. Of interest, a clear time of day effect has been described in serval cardiac surgery studies, noting higher blood transfusion rates and even increased mortality [19, 20]. With this in mind, our institution’s avoidance of late starts in the Cardiac service line (5% of total cases) in understandable. 

The limitations of this work include its single institutional, retrospective nature and the lack of outcome data with regard to readmission, and surgeon-specific post-operative management guidelines. While adoption of universal parameters for urinary catheter removal and post-operative mobilization have likely served to mitigate some of these issues, their impact remains uncertain in the current data set. In addition, the surgical scheduling of elective cases is a department-specific process rather than centralized with preservation of service line block time. Hence, the possibility of service line-specific bias in case-mix scheduling could be present within these data. Similarly, possible differences in weekend discharge resources were not considered in cases performed on Fridays, as day of the week effect analysis was not performed. 

Overall, these data support the concept that late start (after 3 PM) elective surgical cases result in an increase in median LOS for patients, specifically those undergoing Orthopedic and Neurosurgical cases. From a cost perspective, this increase LOS by one day represents a total of 487 inpatient hospitalization days for which there is no increased revenue generation, and a decrease in occupancy capacity. In an era of emphasizing increased value and expenditure capitation, additional attention spent on optimizing the scheduling of elective surgical cases has the potential for improvement for both patients and healthcare delivery systems. To that end, a centralized approach to the scheduling of elective surgical cases and recognition of the need for cases to be started in a timely manner during daytime business hours may offer an improved method for decreasing unnecessary LOS increases over time. 

## Conclusions

Surgical scheduling of elective procedures with planned inpatient hospitalizations to start after 3 PM has the potential to lengthen stay. Such increased length of stay may result in opportunity loss for elective procedure margin, indicating a role for consideration of developing institutional strategies for scheduling optimization. In the post-COVID-19 era and with the emergence of value-based payments, both patient demand and institutional fiscal requirements stand to stress an already imperfect system. In reflecting on strategies for optimizing throughput, we believe that examination of surgical start time may provide a value benefit for patients and providers. 
